# Exosomes derived from siRNA against GRP78 modified bone-marrow-derived mesenchymal stem cells suppress Sorafenib resistance in hepatocellular carcinoma

**DOI:** 10.1186/s12951-018-0429-z

**Published:** 2018-12-20

**Authors:** Hongdan Li, Cheng Yang, Yijie Shi, Liang Zhao

**Affiliations:** 1 Life Science Institute, Jinzhou Medical University, Jinzhou, 121000 People’s Republic of China; 2 Department of General Surgery 2, Central Hospital of Jinzhou City, Jinzhou, 121000 People’s Republic of China; 3 School of Pharmacy, Jinzhou Medical University, Jinzhou, 121000 People’s Republic of China

**Keywords:** Exosome, GRP78, Sorafenib, Growth, Metastasis, Hepatocellular carcinoma

## Abstract

**Background:**

Sorafenib is an effective clinical drug in therapy of hepatocellular carcinoma, having led to improved prognosis in hepatocellular carcinoma patients. However acquired resistance is still being encountered. So, it is urgently to develop alternative strategies to overcome drug resistance. Exosomes can be modified with a variety of molecules, thereby acting as a vehicle for the delivery of therapeutic agents. The GRP78 is overexpressed in Sorafenib resistant cancer cells compared to Sorafenib sensitive cancer cells and thus is able to act as a target for therapy of hepatocellular carcinoma.

**Results:**

In this study, we modified BM-MSCs to express the exosomal siGRP78. And we show that siGRP78 modified exosomes combined with Sorafenib is able to target GRP78 in hepatocellular carcinoma cells and inhibit the growth and invasion of the cancer cells in vitro. Further, siGRP78 modified exosomes combined with Sorafenib also inhibit the growth and metastasis of the cancer cells in vivo.

**Conclusions:**

siGRP78 modified exosomes could sensitize Sorafenib resistant cancer cells to Sorafenib and reverse the drug resistance.

**Electronic supplementary material:**

The online version of this article (10.1186/s12951-018-0429-z) contains supplementary material, which is available to authorized users.

## Background

Hepatocellular carcinoma (HCC) is the sixth most common tumor and the second most frequent cause of cancer death worldwide [1, 2]. Nowadays, HCC presents a high incidence and mortality. Although many treatment have improved and diagnostic standardization has been better [3], improved overall survival of patients is difficult.

Sorafenib [4] is an oral multikinase inhibitor which inhibits HCC proliferation and increases apoptosis by inhibiting the serine-threonine kinases BRAF and CRAF and the receptor tyrosine kinases vascular endothelial growth factors receptors (VEGFRs) and platelet-derived growth factor receptor β (PDGFR-β) [5]. Until now, Sorafenib is still the only FDA approved systemic drug for the treatment of unresectable advanced HCC. However, acquired resistance to Sorafenib in HCC patients is a common phenomenon and limits its clinical application [6–8].

Grp78 is overexpressed in many tumors and has been linked to the progression of many human cancers including colon cancer [9], lung cancer [10], gastric cancer [11], breast cancer [12], Hepatocellular carcinoma [13]. Our research group not only find GRP78 play important roles in HCC, but also find GRP78 promotes the drug resistance to Sorafenib [5, 14]. As a strategy for targeting drug resistance, the application of nucleic acid-based inhibitors of gene expression, such as RNA interference (RNAi), has been proposed in the treatment of many tumors [15–19]. And with the development of exosomes, researchers find exosomes is a therapeutic approach to delivery siRNA and some other factors [20, 21].

Exosomes are small nanometer-sized (40–100 nm) vesicles of endocytic origin. They are initially formed within the endosomal compartment and, subsequently secreted when a multi-vesicular body (MVB) fuses with the plasma membrane [22, 23]. These vesicles are released by any type of cells including cancer cells [24]. It was recently reported that exosomes also contain siRNA and microRNA that are transferred to target cancer cells, where they can be translated or mediate RNA silencing [25, 26]. In intercellular communication, exosomes have been considered messengers. Furthermore, exosomes have a complex protein membrane composition that contributes to efficient cellular uptake [27, 28]. Exosomes are an extremely promising therapeutic tool for numerous diseases given their ability to shuttle small molecules between cells. In particular, exosomes avoid immune recognition and clearance compared to exogenous nanovesicles [23]. They have been used widely, such as diabetes [29], cartilage tissue regeneration [30], stroke [31], tumors [32] and et al.

**Fig. 1:**
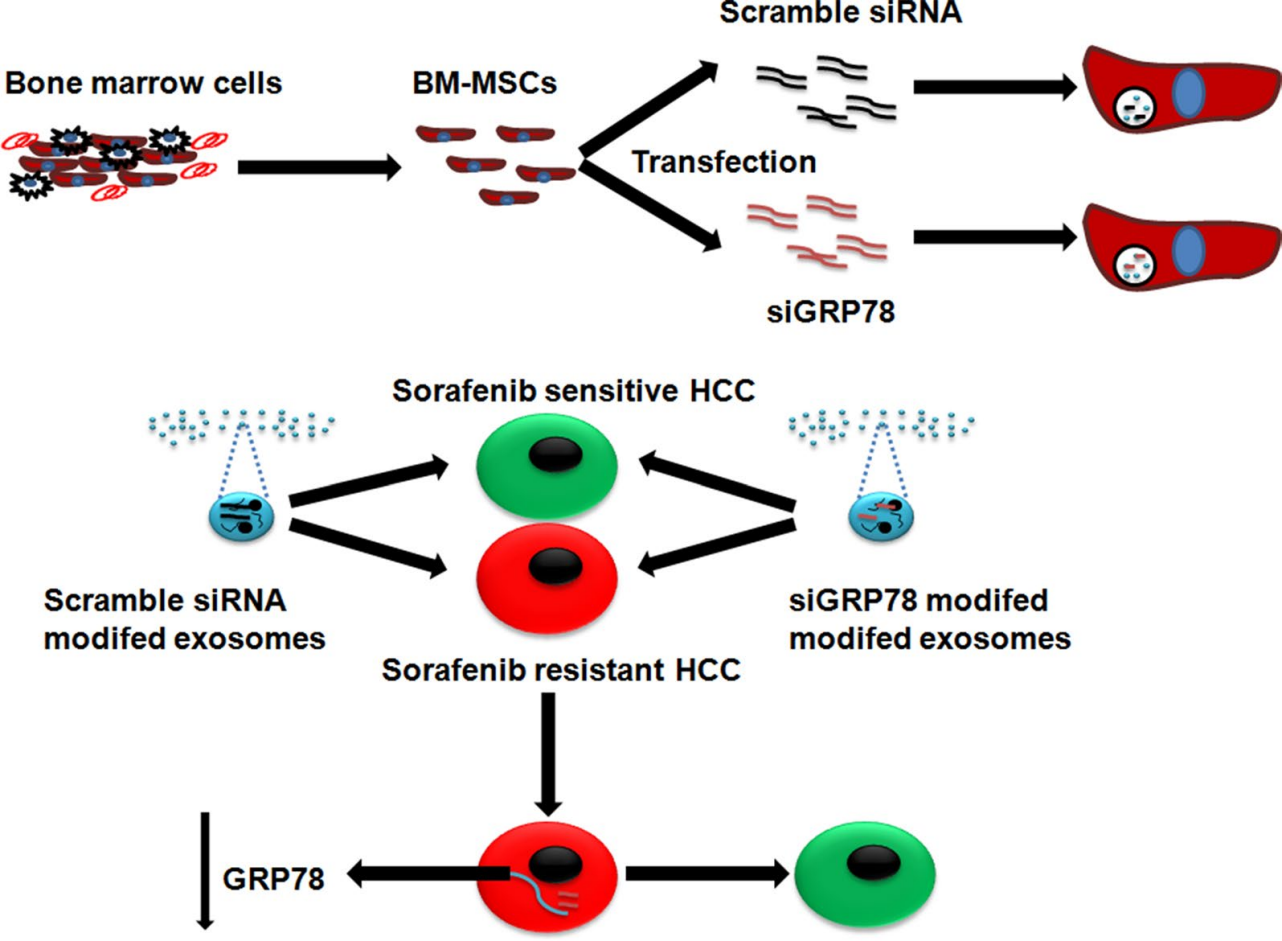
Schematic representation of exosomes derived from BM-MSCs. BM-MSCs were transfected with scramble siRNA and siGRP78 to generate scramble siRNA modified exosomes and siGRP78 modified exosomes. The effect of modified exosomes were tested on Sorafenib sensitive or resistant HCC cells

At present, many researchers used bone marrow mesenchymal stem cells (BM-MSCs) as a tool to gain modified exosomes for its low immunogens [33]. In this study, we generated modified BM-MSCs derived exosomes able to deliver GRP78 siRNA to hepatocellular carcinoma cells to overcome pharmacological resistance of Sorafenib (Fig. 1).

## Results

### Characterization of siRNA against GRP78 modified BM-MSCs

**Fig. 2:**
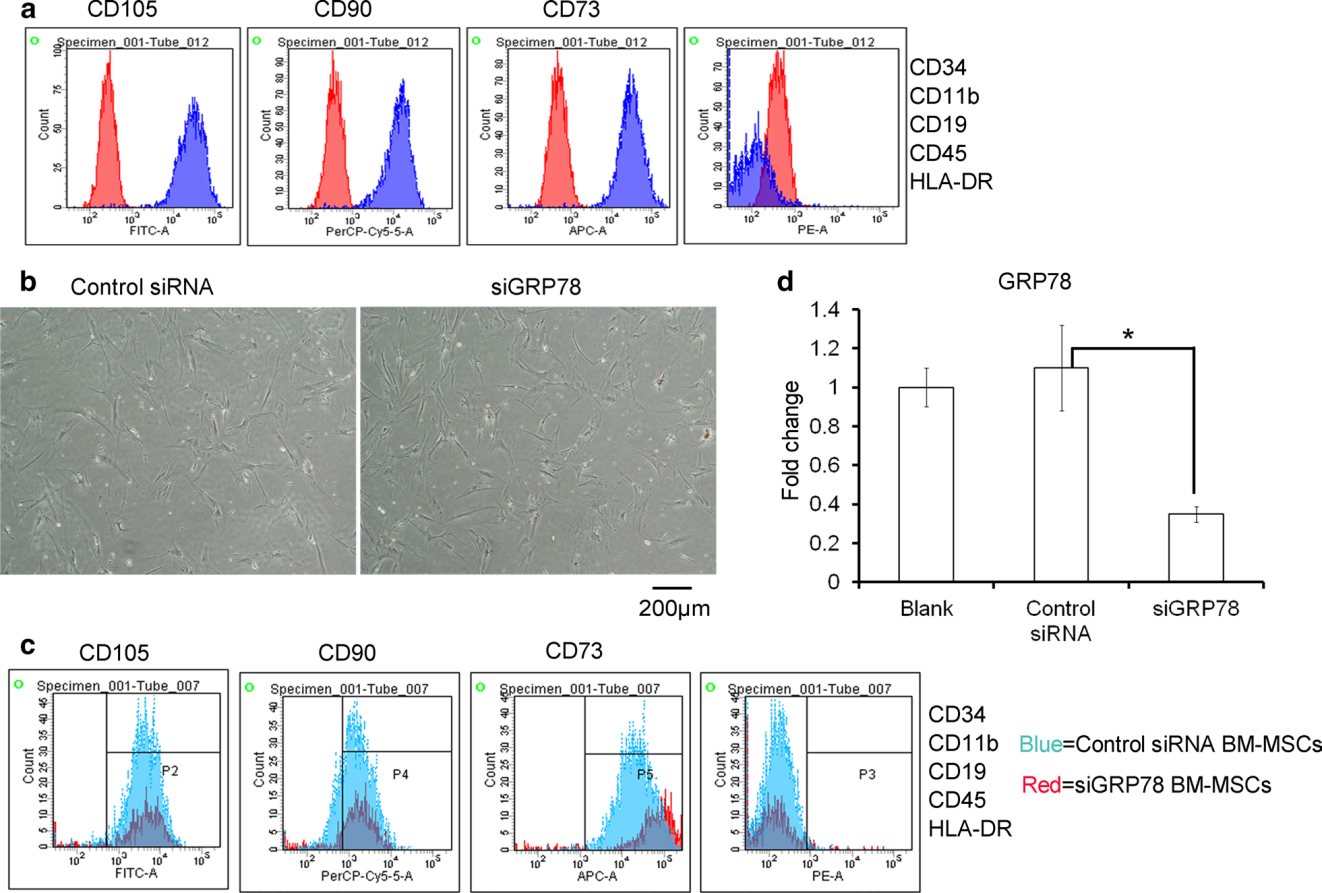
Isolation and Characterization of siGRP78 modified MSCs derived from human bone marrow. **a** Flow cytometric analysis showed BM-MSCs were positive for mesenchymal lineage markers (CD73, CD90 and CD105), negative for hematopoietic and endothelial markers (CD34, CD11b, CD19, CD45), and negative for HLA-DR. **b** Representative morphology of BM-MSCs. **c** Down-regulating the expression of GRP78 in BM-MSCs do not influence the stemness trait of MSC. Blue was the control siRNA; red was the siGRP78. **d** qPCR showed GRP78 was down-regulated in the siGRP78 transfected BM-MSCs compared with control siRNA (scramble siRNA)

To produce siGRP78 expressing exosomes, we isolated BM-MSCs (Fig. 2a) and transfected with siGRP78 or control siRNA into BM-MSCs (Fig. 2b). Then, we identified the expression of GRP78 in these cells expressed siGRP78 as shown by qPCR analysis. The flow cytometer results showed that GRP78 did not influence the stemness trait of BM-MSCs (Fig. 2c). qPCR results showed GRP78 was down-regulated in the siGRP78 transfected BM-MSCs (Fig. 2d).

### Characterization of Sorafenib resistant HCC cells

To establish Sorafenib resistant cancer cells, we exposed HCC cells HepG2 and PLC to increasing concentrations of Sorafenib. From the MTT assay, we found the IC50 of Sorafenib in HepG2 was about 10 µM, and PLC 12.5 µM, however the IC50 in HepG2-SR and PLC-SR cells was more than 20 µM (Additional file 1: Figure S1A). Western blot showed GRP78 is overexpressed in SR cells. qPCR showed the same result with Western blot, GRP78 mRNA expressed higher in SR cells than in control cells. Therefore, we selected 10 µM Sorafenib to treat HepG2 and 12.5 µM Sorafenib to treat PLC cells in our further assays.

### Characterization of exosomes from siRNA against GRP78 modified BM-MSCs

**Fig. 3:**
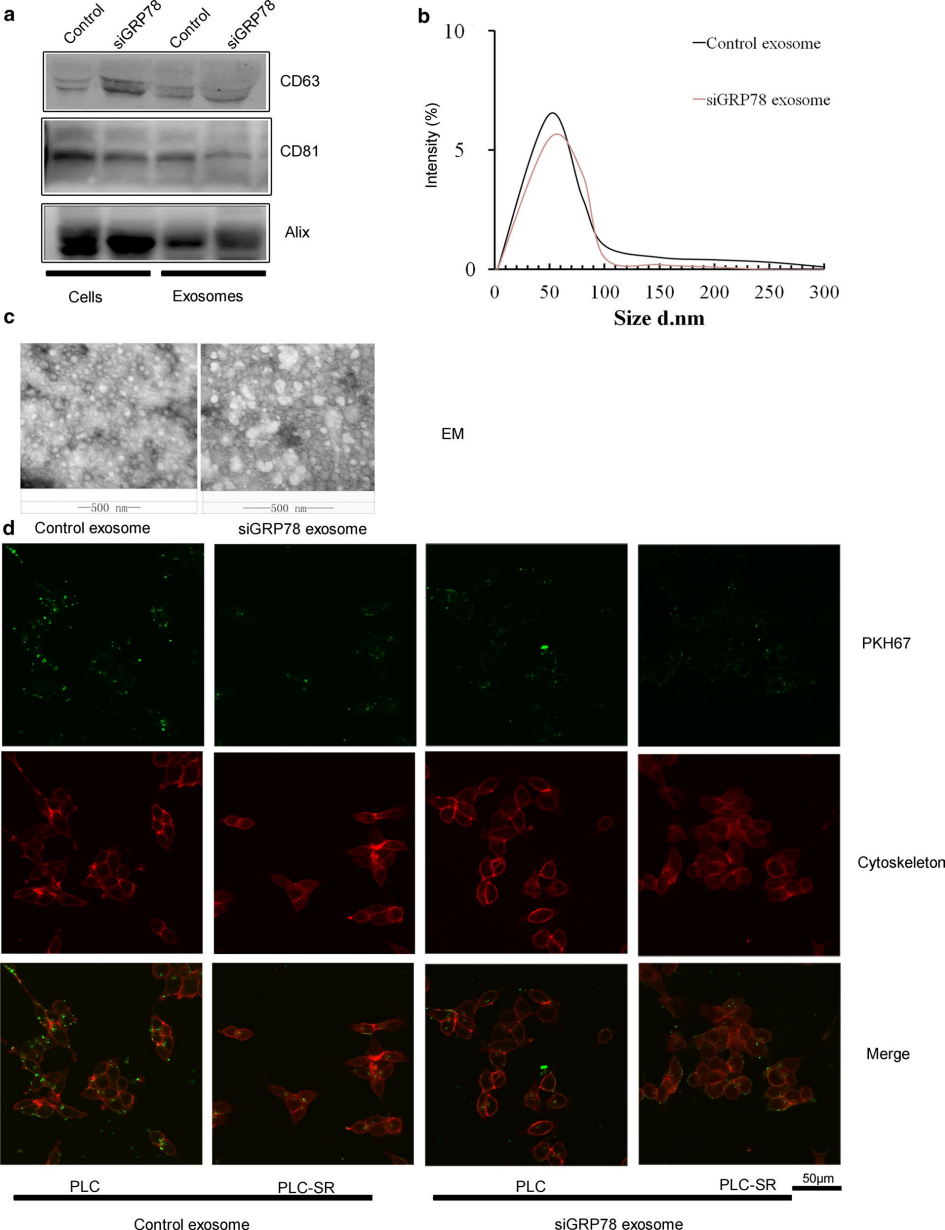
Generation and characterization of siGRP78 modified exosomes. **a** Western blot showed the exosomes from transfected or not with siGRP78. Protein levels of the two exosomes, CD63, CD81 and Alix were evaluated. **b** Exosome size distribution was determined by NTA. **c** Electron microscopy showed the morphology of BM-MSCs-derived exosomes. Scale bar = 500 nm. **d** Confocal microscopy showed PLC cells treated with 10 μg/ml of control exosomes derived from BM-MSCs and siGRP78 modified exosomes from BM-MSCs. Cytoskeleton were stained with phalloidin-TRITC (red); exosomes were labeled with PKH67 (green). Scale bar = 50 μm

We transfected BM-MSCs with control siRNAs (scrambled siRNA) and siGRP78, exosomes were isolated from the conditioned medium 24 h after transfection and used for our further studies. To characterize the siGRP78 (siRNA against GRP78) modified exosomes, firstly, we examed the expression of exosomal markers [34]: Alix, CD81 and CD63, which are all expressed in the modified exosomes (Fig. 2a). Then, we detected exosome size distribution (ranging between 4 and 120 nm) by nanoparticle tracking analysis (NTA), and morphology by electron microscopy (EM) (Fig. 3b, c). By transmission electron microscopy, we determined BM-MSCs-derived exosomes were about 50 to 130 nm in width and physically homogeneous (Fig. 3c). Totally, the data suggest that exosome modification does not alter their size or surface markers. To quantify the loading efficiency of siGRP78 in exosomes from BM-MSCs, we used RT-PCR and found that 1% of the siRNA was retained in the exosomes after transfection.

To determine whether BM-MSCs—derived exosomes, either expressing siGRP78 or not, could be internalized by Sorafenib sensitive or resistant HCC cells, exosomes were labeled with the lipophilic dye PKH67. HepG2 and PLC cells, and their resistant cells (SR), treated at 37 °C with 10 μg/ml of exosomes for 3 h, internalized exosomes as shown in Fig. 3d and in Additional file 1: Figure S2. The results showed that siGRP78 modified exosomes could be internalized by all the cells. So, siGRP78 modified exosomes did not influence the internalization of exosomes.

### siGRP78 modified exosomes combined with Sorafenib inhibit the growth of HCC

As lack of an appropriate delivery systems, the RNA-based therapy of HCC has been hampered in clinic. Here, we examined the possibility of loading exosomes with GRP78 specific siRNA to test their functional activity towards Sorafenib sensitive and resistant HCC cells. To test whether siGRP modified exosomes showed functional activity in inhibiting Sorafenib sensitive and resistant HCC cell growth, we treated Sorafenib-sensitive or resistant HepG2 or PLC cells for 48 h with 0.1, 0.5, 1 or 10 μg/ml of exosomes with scrambled siRNA or with siGRP78 and combined with or without Sorafenib (HepG2 was 10 µM, and PLC was 12.5 µM).

**Fig. 4:**
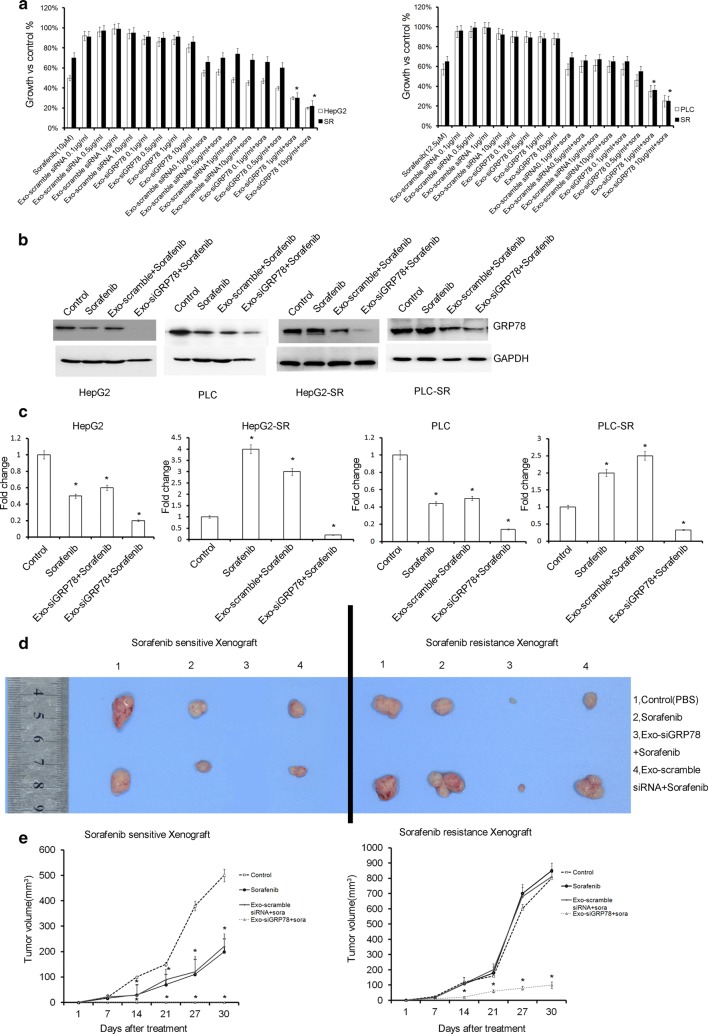
Effects of siGRP78 modified exosomes on the growth of Sorafenib sensitive and resistant cancer cells. **a** HepG2 (left panel) and PLC (right panel) growth was measured by MTT assay after 48 h, (Sorafenib; 0.1, 0.5, 1 or 10 μg/ml of Exo-scrambled siRNA or Exo-siGRP78 exosomes; and 0.1, 0.5, 1 or 10 μg/ml of Exo-scrambled siRNA or Exo-siGRP78 modified exosomes combined with or without Sorafenib. The values were plotted as % of growth vs Ctrl (untreated cells). Each point represents the mean ± SEM of three independent experiments (*p < 0. 05, versus Sorafenib treatment). **b** Western blot analysis was performed on HepG2 and PLC cell lines, and their resistant cells treated for 48 h with Sorafenib, Sorafenib + scramble siRNA modified exosomes derived from BM-MSCs or siGRP78 modified exosomes + Sorafenib derived from BM-MSCs. Protein levels of GRP78 were evaluated. GAPDH as internal control. **c** qPCR showed the expression of GRP78 in HepG2 and PLC cell lines, and their resistant cells treated for 48 h with Sorafenib, Sorafenib + scramble siRNA modified exosomes or Sorafenib + siGRP78 modified exosomes derived from BM-MSCs. GAPDH as internal control. **d** The Subcutaneous orthotopic tumour growth in vivo assay showed the tumor size after different treatments (Control, Sorafenib, Exo-scramble siRNA + Sora and Exo-siGRP78 + Sora). Exo-siGRP78 + Sora significantly inhibited the the growth of sensitive or resistant cancer cells. (1) Control (PBS); (2) Sorafenib; (3) Exo-siGRP78 + Sora; (4) Exo-scramble siRNA + Sora, (n = 5 for each group, injections at 25 mg/kg, q.o.d). **e** The median tumor volume showed the antitumor efficacy of the different treatments (Control, Sorafenib, Exo-scramble siRNA + Sora and Exo-siGRP78 + Sora). Significant differences in terms of tumor volume were observed from day 14, Exo-siGRP78 + Sora versus Exo-scramble siRNA + Sora (*P < 0.05)

From Fig. 4a, we observed dose dependent reduced viability of the four cell lines treated with Sorafenib and siGRP78-modified exosomes 0.1, 0.5, 1 or 10 μg/ml of exosomes (*P *> 0.05). As expected, Sorafenib treatment did not inhibit HepG2-SR (Sorafenib resistance) and PLC-SR cell growth. No differences were found in scrambled siRNA-modified exosomes compared to controls. siGRP78 modified exosomes inhibit the growth of sensitive and resistant HCC slightly. We also found that 10 μg/ml of siGRP78-modified exosomes was the most effective concentration in HCC. So, we used 10 μg/ml of siGRP78-modified exosomes for our further experiments.

To explore the relationship between anti-proliferative effect and GRP78 in Sorafenib resistance, Sorafenib sensitive or resistant cells treated with siGRP78-modified exosomes or scramble siRNA exosomes were added Sorafenib and subjected to immunoblotting and qPCR to detect the expression of GRP78. As shown in Fig. 4b, c, the treatment of HCC cells with siGRP78 modified exosomes was able to decrease the expression of GRP78 in all cells. And Sorafenib only inhibited GRP78 in sensitive HCC. In Sorafenib resistant HCC, Sorafenib could not inhibit GRP78 expression, inversely, promoted the mRNA expression of GRP78.

The ability of siGRP78 modified exosomes combined with Sorafenib to reduce HCC growth was also tested in an in vivo tumor xenograft model. PLC and PLC-SR cells (1 × 10^7^) were inoculated subcutaneously in Balb/c nu/nu mice; 1 week post cell injection, mice were injected around the tumor q.o.d with vehicle (PBS), Sorafenib (25 mg/kg) and 100 μg of exosomes released by BM-MSCs (Exo-scramble siRNA or Exo-siGRP78) with Sorafenib (25 mg/kg). After 1 month, mice were sacrificed and the tumors removed. Figure 4d and Additional file 1: Figure S3 showed that tumor growth of PLC was reduced in mice treated with Sorafenib, and no tumor were found in mice treated with siGRP78 modified exosomes combined with Sorafenib. Correspondingly, in PLC-SR, obvious reduction in tumor size was observed in mice treated with siGRP78 modified exosomes combined with Sorafenib. There were no statistically significant differences between mice treated with control (scramble siRNA) exosomes combined with Sorafenib and Sorafenib treatment. Additional file 1: Figure S3 showed the final tumor weight of the tumors. Figure 4e showed that tumor growth of sensitive and resistant cells was reduced in mice treated with Sorafenib and siGRP78 modified exosomes. As expected, Sorafenib with or without scramble siRNA modified exosomes could not inhibit the tumor growth of resistant cancer cells. Compared with sensitive cells, Sorafenib resistant cells were resistant to Sorafenib.

Together, siGRP78 modified exosomes facilitated the sensitivity of HCC to Sorafenib and reversed the Sorafenib resistance in HCC.

### siGRP78 modified exosomes combined with Sorafenib inhibit the invasion of HCC in vitro

**Fig. 5:**
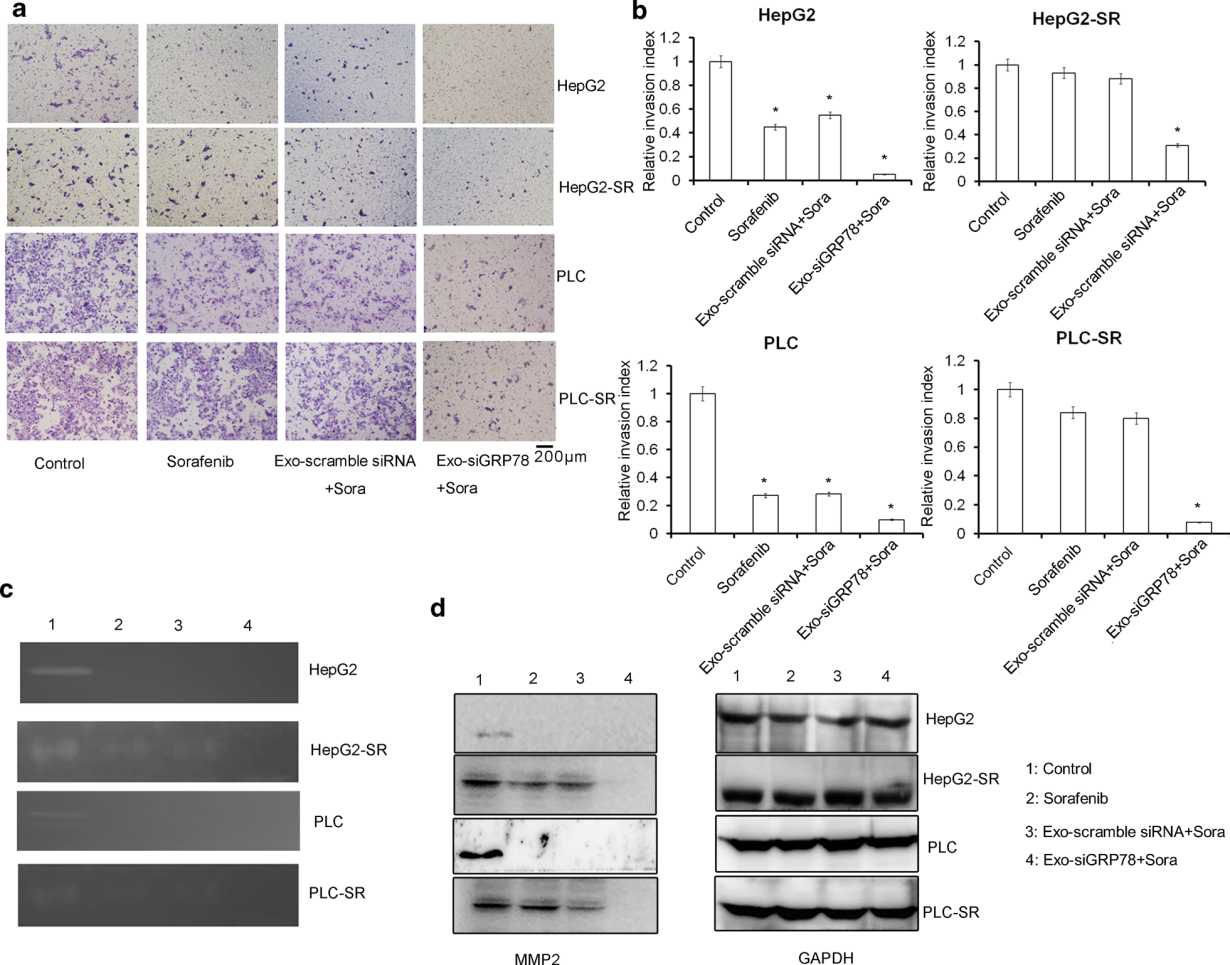
The effect of siGRP78 modified exosomes combined with Sorafenib on the invasion of HCC. **a** Transwell assay showed the cell invasive ability in sensitive and resistant cancer cells with different treatments (Control, Sorafenib, Exo-scramble siRNA + Sora and Exo-siGRP78 + Sora). Scale bar = 200 μm. **b** The statistical analysis showed the differences of different treatments normalized to the invasion index of control cells. Data are mean ± SEM (n = 3, *P < 0.05). **c** Gelatin zymography showed extracellular matrix degradation of HCC by different treatments (1: Control, 2: Sorafenib, 3: Exo-scramble siRNA + Sora and 4: Exo-siGRP78 + Sora). **d** Western blot showed the expression of MMP2 in Sorafenib sensitive and resistant cancer cells by different treatments. (1: Control, 2: Sorafenib, 3: Exo-scramble siRNA + Sora and 4: Exo-siGRP78 + Sora)

To test whether siGRP modified exosomes could inhibit the invasion of Sorafenib sensitive and resistant HCC, we treated Sorafenib-sensitive or resistant HepG2 or PLC cells for 48 h with the four treatments (Control, Sorafenib, Sorafenib with scramble siRNA exosomes, Sorafenib with siGRP78 exosomes). The dose of Sorafenib uesd in HepG2 was 10 µM, and PLC was 12.5 µM. Then, we added the cells in the up layers. After 24 h, we found Sorafenib could not inhibit the invasion of SR cells, and siGRP78 modified exosomes combined with Sorafenib inhibited the invasion of SR (Fig. 5a). No differences compared to Sorafenib were observed in scrambled siRNA modified exosomes. Figure 5b showed the statistic analysis (*P < 0.05). To show the extracellular matrix degradation of HCC, we used zymography assay and found Sorafenib inhibited matrix degradation in sensitive cells. However, Sorafenib could not inhibit matrix degradation of resistant cells. siGRP78 modified exosomes combined with Sorafenib inhibited the ability of matrix degradation in sensitive and resistant cells (Fig. 5c). Figure 5d also showed siGRP78 modified exosomes combined with Sorafenib inhibited the expression of MMP2 in sensitive and resistant cells. Correspondingly, Sorafenib could not inhibit the expression of MMP2 in resistant cells, and inhibited MMP2 in sensitive cells. The scramble siRNA exosomes could not sensitize SR cells to Sorafenib.

### siGRP78 modified exosomes combined with Sorafenib inhibit the metastasis of Sorafenib resistant cells in vivo

**Fig. 6:**
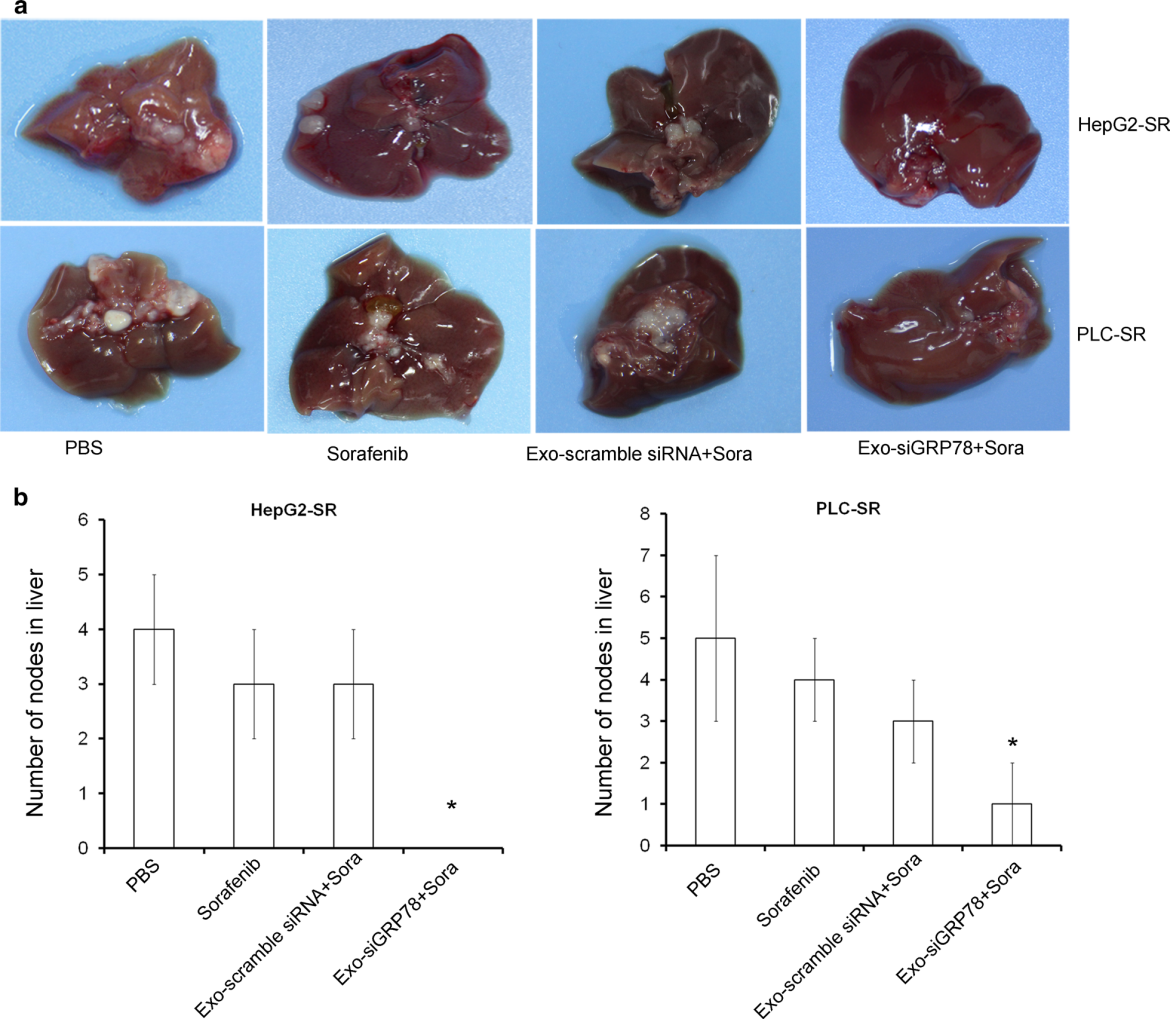
The effect of siGRP78 modified exosomes on the metastasis of Sorafenib resistant cancer cells. **a** The orthotopic metastasis model showed the tumor metastasis in liver by different treatments (Control, Sorafenib, Exo-scramble siRNA + Sora and Exo-siGRP78 + Sora). **b** Statistic analysis showed the number of tumor nodes in liver of mice (*P < 0.05, versus Sorafenib treatment)

To investigate the role of siGRP78 modified exosomes on SR cells metastasis, HepG2-SR (1 × 10^7^/100 μl) and PLC-SR (1 × 10^7^/100 μl) cells were infected into the tail vein of Balb/c nu/nu mice. The 10 mice were randomly separated into 2 groups. One week after cell injection, mice were treated intraperitoneally q.o.d with vehicle (PBS), Sorafenib (25 mg/kg), 100 μg of scramble siRNA modified exosomes + Sora and 100 μg of siGRP78 modified exosomes + Sora. One month later, mice were sacrificed. We found that the treatment of mice bearing Sorafenib-resistant cells with siGRP78 modified exosomes determined less tumor metastasis in liver, compared to control mice (PBS) and to mice treated with scramble siRNA exosomes. No differences were observed in mice treated with exosomes containing scrambled siRNAs combined with Sorafenib compared to Sorafenib group (Fig. 6a). Figure 6b analyzed the number of tumor nodes in liver.

The data suggest that siGRP78 modified exosomes sensitize SR cells to Sorafenib in HCC metastasis.

## Discussion

Although Sorafenib as tyrosine kinase inhibitor has revolutionized treatment and improved prognosis in HCC patients, the development of pharmacological resistance still remains a tricky problem [35]. One of the effective methods is to transfect a short interfering RNA (siRNA) to downregulate the expression of aberrant protein responsible for this drug resistance [19]. Although this approach is highly viable, it is difficult to find an effective target gene. Thereby, for clinical application of siRNAs, the stability and efficiency of delivery system is also a key challenge.

In this study, we propose a new approach to convey siRNA against GRP78, which plays vital roles in the process of Sorafenib resistance. BM-MSCs derived exosomes were used as a delivery system. BM-MSCs are a kind of adult stem cells, which have fine histocompatibility as tool cells. At present, the BM-MSCs are more and more popular in the therapy of diseases. Exosomes are natural carriers between cells in physiological state and also transfer chemotherapeutic drugs into tumors. Kim et al. used exosomes to deliver paclitaxel or doxorubicin in order to overcome multiple drug resistance in lung cancer.

Based on the previous researches, we tried to use siGRP78 modified exosomes from BM-MSCs to treat HepG2 and PLC cells or their resistant cells. First, we isolated BM-MSCs and transfected siGRP78 and scramble siRNA into the cells. And we found the exosomes could express siGRP78 and do not change the stemness of the BM-MSCs (Fig. 2). Then, we analyzed the exosomes from siGRP78 modified exosomes compared with scramble siRNA by western blot, NTA and EM, and demonstrated that the siGRP78 could not change the molecular markers, morphology and size of the exosomes of BM-MSCs (Fig. 3). In addition, confocal microscopy analysis showed that the sensitive and resistant cancer cells internalize exosomes with or without siGRP78. This confirm the efficiency of siGRP78 or scramble siRNA delivery approach (Fig. 3d and Figure S2). It is possible to use exosomes in the delivery of siGRP78 to HCC cells. To demonstrate the effect of siGRP78 on the sensitive and resistant cancer cells, we employed MTT assay and found siGRP78 modified exosomes combined with Sorafenib inhibited the growth of both sensitive and resistant cancer cells. As expected, Sorafenib could inhibit the growth of sensitive cancer cells, but could not inhibit the growth of resistant cancer cells (Fig. 4a). To deeply explain GRP78 is involved in Sorafenib resistance, we detected the expression of GRP78 in all the cells of different treatments. And the results showed siGRP78 modified exosomes inhibit the expression of GRP78 in Sorafenib resistant cancer cells by Western blot. Also, in Sorafenib sensitive cancer cells, siGRP78 modified exosomes inhibited the expression of GRP78 (Fig. 4b). qPCR results showed the consistant results with Western blot (Fig. 4c). In vivo results showed the siGRP78 modified exosomes combined with Sorafenib obviously inhibit the growth of Sorafenib resistant cells. In Sorafenib sensitive cells group, there are no tumor growth (Fig. 4d, e). Finally, Transwell assay showed siGRP78 modified exosomes combined with Sorafenib inhibit the invasive ability. MMP2 and Zymography assay determined siGRP78 modified exosomes suppress the Matrix degradation (Fig. 5). In vivo metastasis model verified the conclusion of siGRP78 modified exosomes sensitize resistant cancer cells to SR (Fig. 6).

Taken together, our data are very promising and provide a rational base for the use of siGRP78 modified exosomes in a Sorafenib resistance therapy approach for use in HCC patients. Nevertheless, siGRP78 modified exosomes combined with Sorafenib could obviously inhibit sensitive HCC tumors. In clinics, we propose siGRP78 modified exosomes combined with Sorafenib together. While focusing on HCC, results from this study might have an impact on other types of tumors, such as colon cancer and gastric cancer, where GRP78 is abundantly expressed.

## Conclusion

In conclusion, siGRP78 modified exosomes combined with Sorafenib are able to target GRP78 in hepatocellular carcinoma cells and inhibit the growth and invasion of the cancer cells in vitro. We demonstrated that exosomal transfer of siGRP78 enhanced chemosensitivity to Sorafenib in drug-resistant hepatocellular carcinoma.

## Materials and methods

### Ethic approval

This study was approval by the Ethic Committee at Jinzhou medical university. The use of the clinical specimens and animal for research purposes was in accordance with the Declaration of Helsinki.

### Isolation and characterization of human mesenchymal stem cells

Bone marrow cells were isolated from femoral head after informed consent from patients undergoing hip-replacement surgery. The marrow were mixed with culture medium (MesenPRO RS™ Medium, Gibco, 12746-012) and isolated by h-BM-MSC isolation kit (TBD). The collected cells were plated in tissue culture flasks without further interference for 2–3 days. The culture medium was depleted by successive changes of culture medium (MesenPRO RS™ Medium, Gibco, 12746-012). A confluent monolayer culture with cells was observed 7 days following initial plating. Human BM-MSCs is characterized by the BD human MSC analysis kit (BD 562245).

### Cell culture

The human Hepatocellular carcinoma cell line HepG2 and PLC were purchased from Cell bank of Chinese Academy of Sciences (Shanghai, PR China). All the cells were maintained in Dulbecco’s modified Eagle’s medium (DMEM, Gibco, Invitrogen, Carlsbad, CA) with 10% fetal bovine serum (FBS, Clark, Houston, TX, USA) and 1% antibiotic–antimycotic solution (Life Technologies, NY, USA).

### Exosomes isolation

Before exosomes collection, the BM-MSC were cultured in culture media containing centrifuged FBS, which was used to remove FBS-derived exosomes. During 24–48 h, the culture medium were collected and prepared for exosomes collection. Exosomes were collected from the medium of 50 ml human BM-MSC cells. The culture media was placed on ice and centrifuged at 800*g* for 10 min to sediment the cells and subsequently was centrifuged at 12,000*g* for 20 min to remove the cellular debris. Exosomes were separated from the supernatant by centrifugation at 100,000*g* for 2 h. The exosome pellet was washed once in a large volume of PBS and re-suspended in 100 μl of PBS (exosomes fraction).

### Exosome fluorescent labeling

Exosomes were also isolated following the same procedure as described above, and for functional assays where exosomes were used, the concentration of total proteins contained in each exosomes pellet was quantified using the BCA assay. Exosomes were labeled with the green fluorescent linker PKH67 (Sigma-Aldrich), as the instruction showed. Briefly, bring the volume of the pellet sample up to 1 mL using Diluent C from the PKH67 kit. Add 6 μl PKH67 dye into each of the 1 ml Diluent C tubes, mix continuously for 30 s by gentle pipetting. Let stand at room temperature for 5 min. Quench by adding 2 ml 10% BSA in PBS. Bring the volume up to 8.5 ml in serum-free media. Make a 0.971 M sucrose solution. Add 1.5 ml of the sucrose solution by pipetting slowly and carefully into the bottom of your tube, making sure not to create turbulence. The exosomes-PKH67 solution will remain on top of a sucrose cushion. Centrifuge at 190,000*g* for 2 h at 2–8 °C. Resuspend the exosome pellet in 1× PBS by gentle pipetting.

### Electron microscopy

Exosomes were adsorbed for 10 min to a carbon coated grid rendered hydrophilic and 20 min fixed with 4% paraformaldehyde, the excess liquid was removed with a filter paper, and samples were stained with 1% uranyl acetate for 30 s. After excess uranyl formate was removed with a filter paper, grids were examined and images were recorded by transmission electron microscope (Japan, Hitachi 7650).

### Nanoparticle tracking analysis

Particle size was measured by dynamic light scattering (Zetasizer Nano ZS; Malvern Instruments, Malvern, UK).

### siRNA transfection

The siRNA sequences against Grp78 were designed by siRNA finder (Ambion, USA) and synthesized by Genechem Corporation (Shanghai, China). The sequences of sense strands of siRNA duplex were as follows: Grp78: 5’-AGACGCUGGAACUAUUGCUUU-3′. BM-MSCs were plated in six-well plate (5 × 10^5^ cells/well), allowed to adhere for 24 h and transfected with siRNA. Transfection of siRNA was performed as lipofectamine 2000 Handbook (Invitrogen). Briefly, the cells were incubated for 4 h with the transfection complex containing 4 μg siRNA. After 4 h, the transfection complex was removed and the cells were incubated in complete growth medium for 48 h. The transfection effect of siRNA was confirmed by qPCR and western blot.

### The preparation and quantification of the modified exosomes

We prepared and quantified as Sander et al. described [36]. After transfection by siRNA or siRNA against GRP78, samples were diluted 10× with PBS and centrifuged at 100,000*g* for 70 min to remove unbound siRNA. RNA was isolated from pellets with TRIzol Reagent according to manufacturer’s recommendations. Reverse transcription of standards and samples was performed in a GeneAmp PCR System 9700 (Applied Biosystems, Foster City, CA) thermocycler using a TaqMan Reverse Transcription Kit, according to manufacturer’s instructions. Each 7.5 μl reverse transcription reaction contained 1 μl of RNA template, 1 mM dNTPs, 1.9 U RNAse Inhibitor, 50 nM reverse stem loop primer and 25 U MultiScribe Reverse Transcriptase in 1× reverse transcription buffer.

Quantitative PCR was performed in 10 μl reactions Amplification curves were analysed with Viia 7 software version 1.2.1. All samples for RT-PCR were prepared in triplicate and each RNA isolate was analysed in duplicate. Using this method, traces of siRNA could still be accurately quantified.

### Transwell assay

Transwell assay was performed at Costar’s 24 well Transwell (Costar #3422). Cells were placed on 96-well-plate, at a concentration of 1 × 10^4^/well. After 24 h, the inserts were inverted and stained with Crystal violet. The number of invade cells were observed and counted using fluorescent microscope. Five fields were randomly chosen and the numbers of penetrated cells were counted.

### MTT assay

To explore the IC50 of HCC, The Hepatocellular carcinoma cells (HepG2 and PLC) were collected and replated into 96-well plate as 10,000 per well, then treated by Sorafenib (0, 2.5, 5, 10, 15, 20, 25 µM).

To explore the effect of siGRP78 modified exosomes on HCC, we treated Sorafenib-sensitive or resistant HepG2 or PLC cells for 48 h with 0.1, 0.5, 1 or 10 μg/ml of exosomes with scrambled siRNA or with siGRP78 and combined with or without Sorafenib, Sorafenib (HepG2 was about 10 µM, and PLC 12.5 µM).

Finally, we added each well with 20 μl of MTT substrate for 4 h; the medium was then removed and 100 μl of DMSO was added. Plates were read at a wavelength of 490 nm, with optical density (OD) reported normalized to blank wells containing only DMSO. We analyzed the relative growth rate as OD (treatment)/OD (control).

### Orthotopic tumour growth in Balb/c Nu Nu mice

Female BALB/c nude mice were purchased from Beijing Vital River Laboratory Animal Technology Co., Ltd. All BALB/c nude mice (4–5 weeks old, female) were maintained in SPF condition. Our animal experiments were approved by the Animal Care Committee.

Subcutaneous model: The 5-week-old BALB/c-nu mice were randomly divided into 4 groups (n = 5 per group). HepG2, HepG2-SR (Sorafenib Resistance) and PLC and PLC-SR cancer cells (1 × 10^7^/0.1 ml PBS) were inoculated subcutaneously into the left and right flank of the nude mice. After 1 week, we injected the drug around the tumors as day 7, 9, 11,…, when tumors were palpable, we treated the tumor with: (1) PBS (Ctrl), (2) Sorafenib (25 mg/kg), (3) Scramble siRNA modified exosomes from BM-MSCs combined with Sorafenib (Exo-scramble siRNA + Sora, 100 μg/mouse + Sorafenib 25 mg/kg), (4) siGRP78 modified exosomes derived exosomes (Exo-siGRP78 + Sora, 100 μg/mouse + Sorafenib 25 mg/kg).

Metastasis model: The 5-week-old BALB/c-nu mice were randomly divided into 4 groups (n = 5 per group). To induce tumor metastasis, 1 × 10^7^ tumor cells were injected into the tail vein of mice. After 1 week, mice were treated intraperitoneally q.o.d with: (1) PBS (Ctrl), (2) Sorafenib (25 mg/kg), (3) Scramble siRNA modified exosomes from BM-MSCs combined with Sorafenib (Exo- scramble siRNA + Sora, 100 μg/mouse + Sorafenib 25 mg/kg), (4) siGRP78 modified exosomes derived exosomes (Exo-siGRP78 + Sora, 100 μg/mouse + Sorafenib 25 mg/kg).

The mice were sacrificed 30 days after inoculation and the tumors were analyzed by tumor weight.

### Western blot analysis

For extraction of total cellular protein, cells were lysed in RIPA buffer with 1% PMSF. Protein concentration was quantified using the BCA kit (Pierce Biotechnology, Inc., Rockford, IL). Proteins were analyzed by SDS-PAGE. The membranes were incubated overnight at 4 °C with the GRP78, MMP2 and GAPDH (1:1000) (Cell signaling technology, Danvas, MA). Thereafter, the membranes were incubated with HRP-labeled anti-rabbit secondary antibodies (1:1000) for 1 h at room temperature. At last, the membrane was visualized by enhanced chemiluminescence kit (Thermo Fisher Scientific Inc., Rockford, IL, USA).

### Gelatin zymography

The Conditioned medium from the HCC cells was collected and concentrated at 2000*g*, 10 min. Equal amounts of protein were loaded and separated by 10% polyacrylamide gel containing 1 g/l gelatin. The gels were re-natured in 2.5% Triton-X-100 with gentle agitation for 30 min at room temperature. The gel was pretreated by developing buffer (5 mM CaCl_2_, 50 mM Tris, and 0.2 mM NaCl, 0.02% Brij35 (pH 7.5)) for 30 min at room temperature, then developed in developing buffer overnight at 37 °C, stained with Coomassie Brilliant Blue R-250 for 30 min and destained with destaining solution. The protease activity was analyzed by gel imaging and analysis system.

### Quantitative real-time PCR assays

mRNAs were isolated with TRIzol reagent (Invitrogen) and reverse transcribed. cDNAs were amplified by RT-PCR. Expression assays were used to quantify the levels of different RNAs as follows:

GRP78(F:TTCAGCCAATTATCAGCAAACTCT;R:TTTTCTGATGTATCCTCTTCACCAGT), GAPDH(F:TGTGGGCATCAATGGATTTGG;R:ACACCATGTATTCCGGGTCAAT). Quantitative PCR was conducted in triplicate at 95 °C for 10 min, followed by 35 cycles of 95 °C for 15 s and 60 °C for 60 s (7300 Fast Real-Time PCR System; Stratagen). Cycle thresholds were normalized to an internal control: U6 rRNA for precursor of miRNA and GAPDH for mRNA assays. The amount of RNA was calculated using the 2−^∆∆CT^ method; the level of expression of RNA was normalized to the adapted internal control (denoted “relative expression”) and, where appropriate, to the level of expression at Control (denoted “fold change”).

### Statistical analysis

All data were expressed as the mean ± SEM. Comparisons between more than two groups were performed by one-way ANOVA. A P-value of < 0.05 was considered statistically significant.

## Additional file

**Additional file 1: Figure S1.** Characterization of Sorafenib resistant HCC cells. **Figure S2.** Characterization of exosomes from siRNA against GRP78 modified BM-MSCs. **Figure S3.** The final tumor weight of the tumors.

## Abbreviations

BM-MSCs: bone marrow mesenchymal stem cells
HCC: hepatocellular carcinoma
Sora: Sorafenib
NTA: nanoparticle tracking analysis
