# Supplementary material for: Exosomes derived from siRNA against GRP78 modified bone-marrow-derived mesenchymal stem cells suppress Sorafenib resistance in hepatocellular carcinoma
Source: J Nanobiotechnology. 2018 Dec 20;16:103. doi: 10.1186/s12951-018-0429-z (PMC6300915; doi:10.1186/s12951-018-0429-z)
Supplement: Supplementary file 1 — Additional file 1: Figure S1. Characterization of Sorafenib resistant HCC cells. Figure S2. Characterization of exosomes from siRNA against GRP78 modified BM-MSCs. Figure S3. The final tumor weight of the tumors. [file 12951_2018_429_MOESM1_ESM.pptx]

## Slide 1
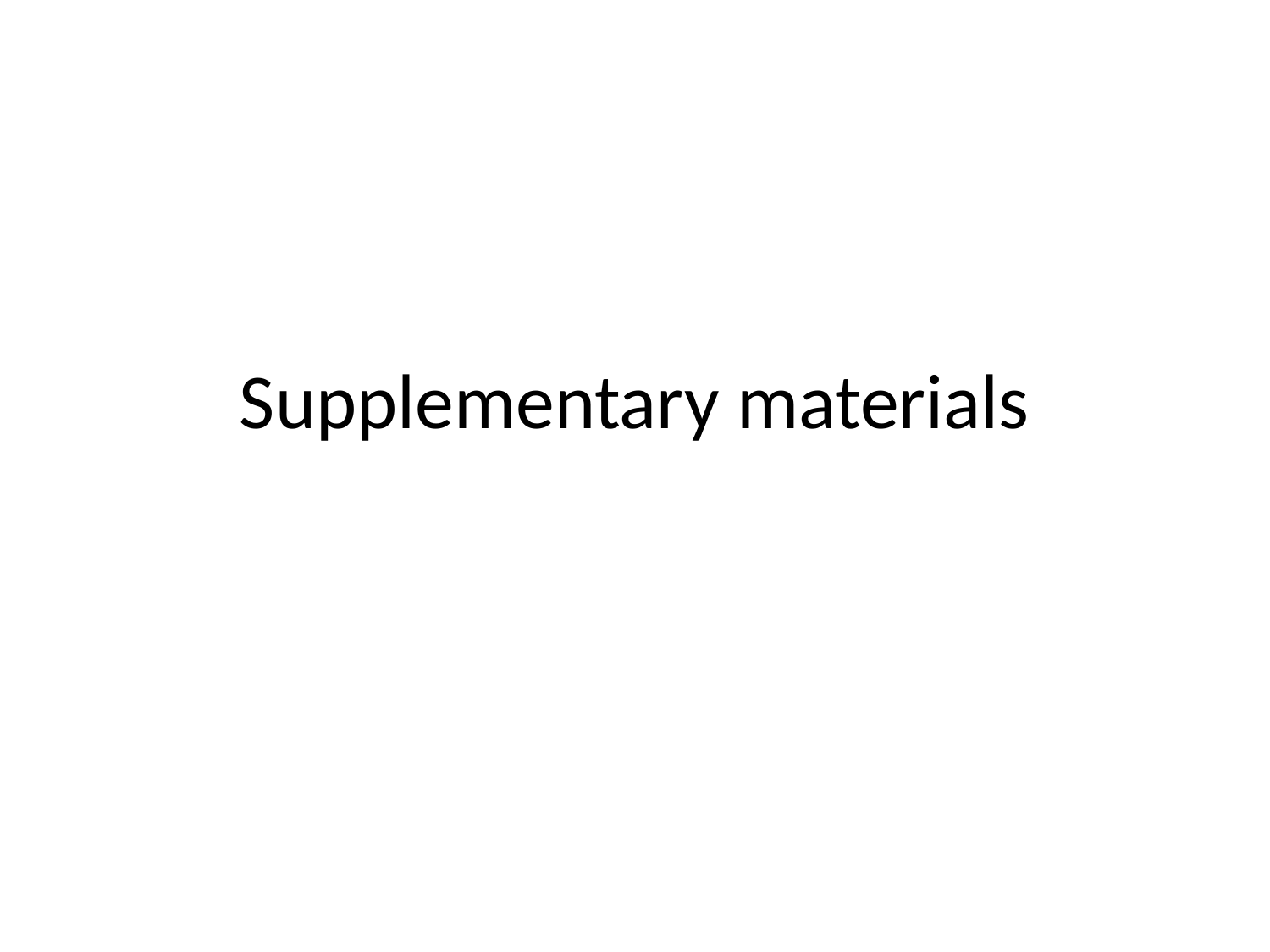

# Supplementary materials

## Slide 2
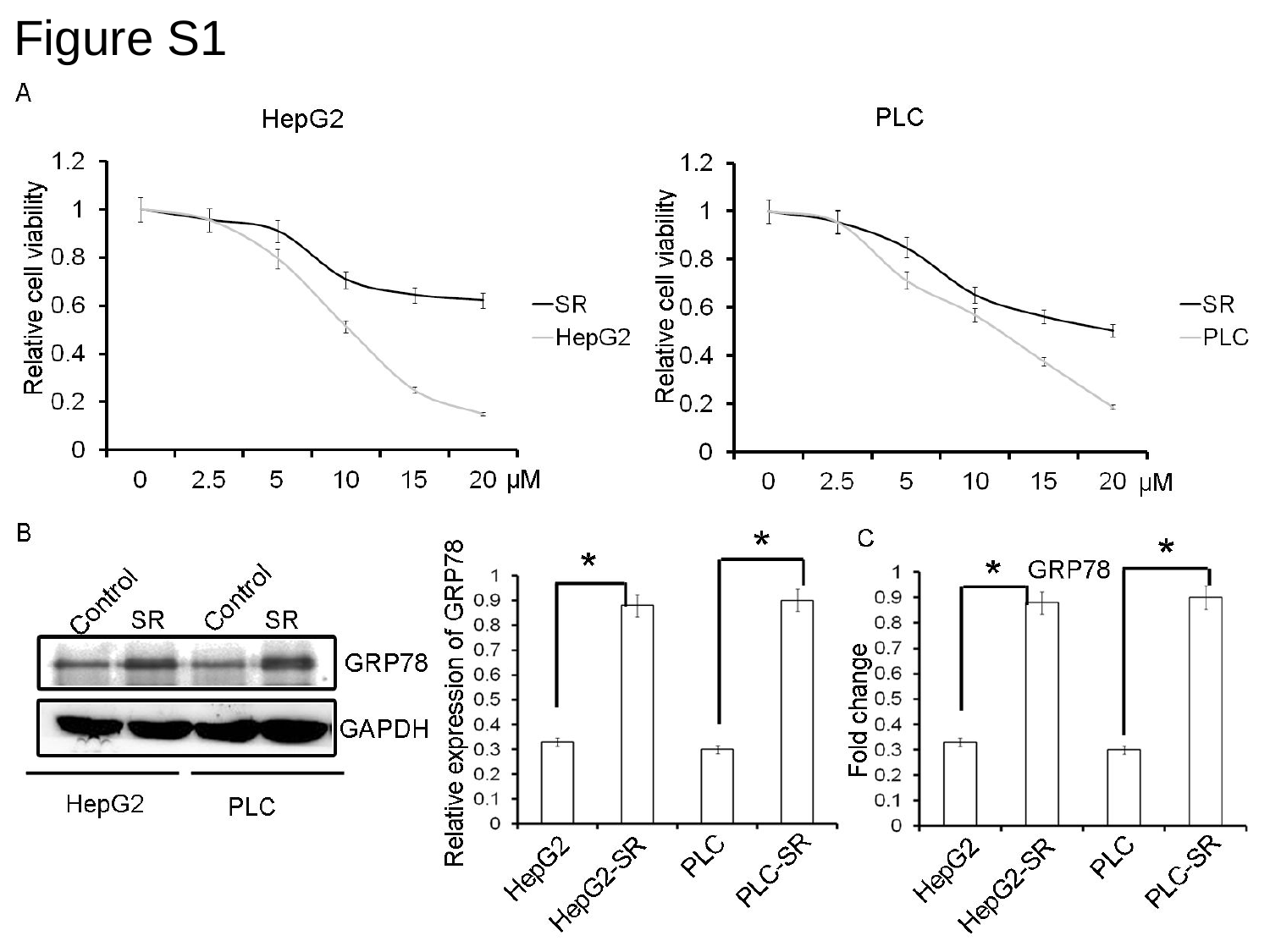

Figure S1

## Slide 3
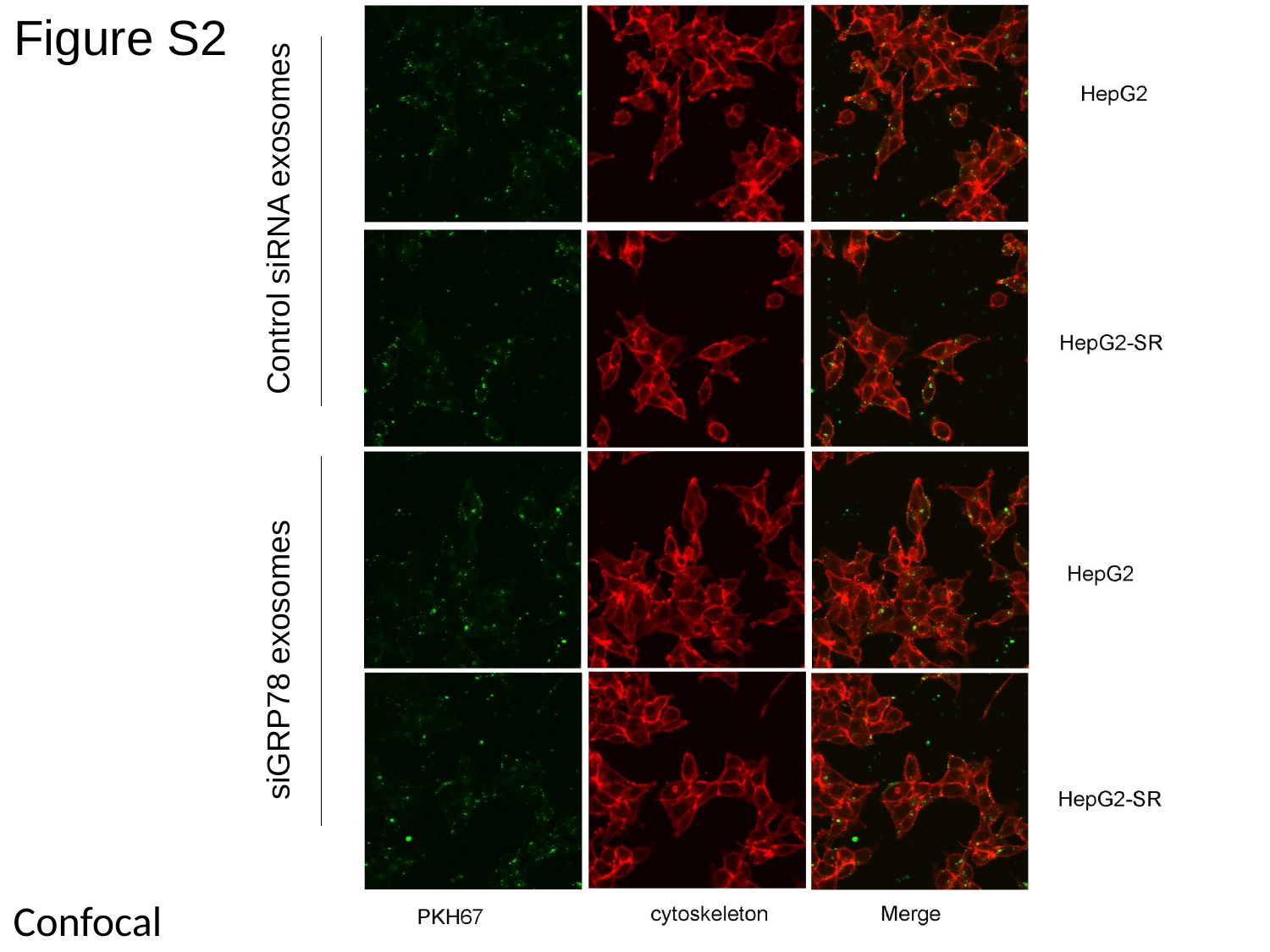

Figure S2
Control siRNA exosomes
siGRP78 exosomes
Confocal

## Slide 4
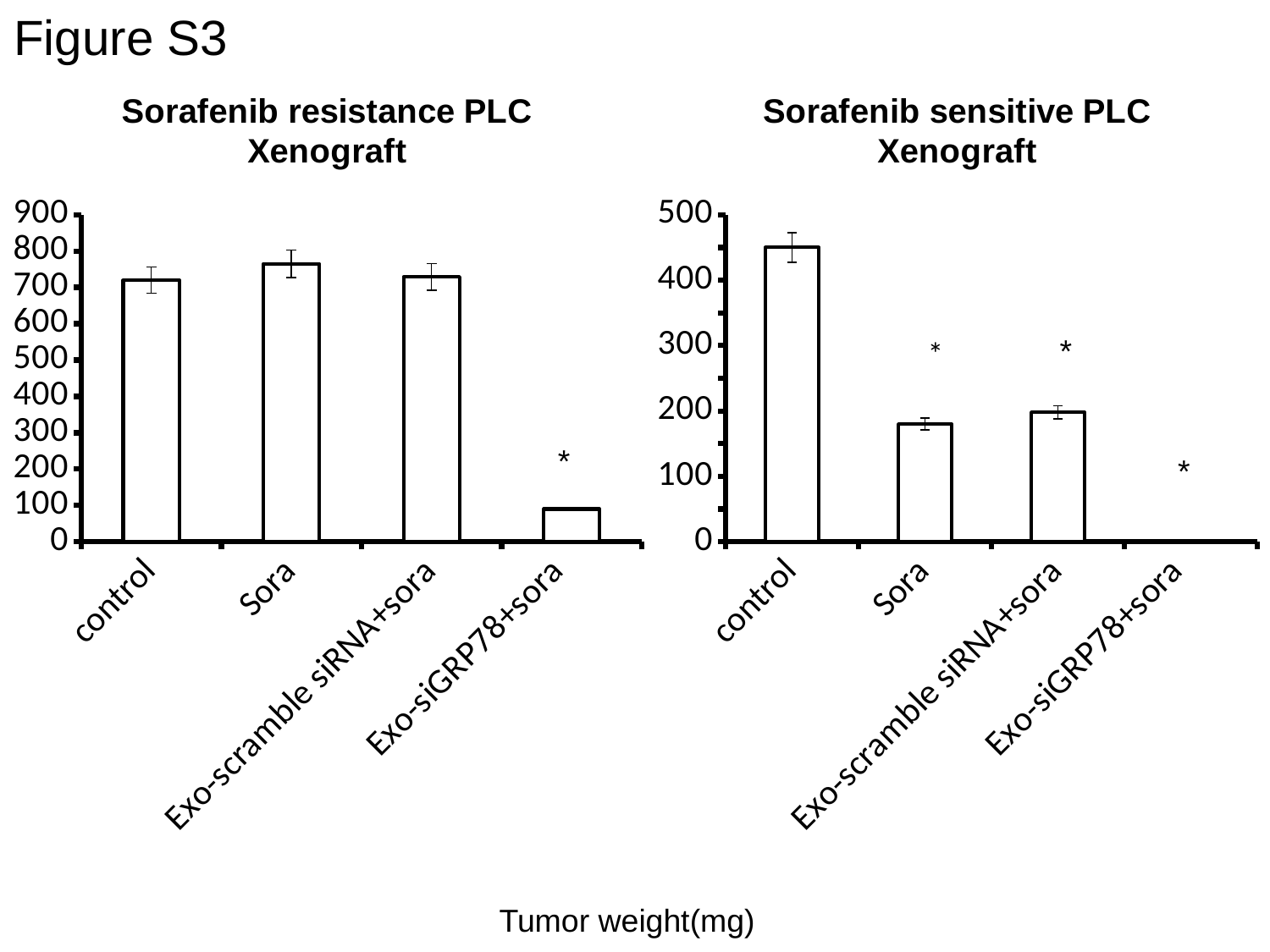

Figure S3
### Chart: Sorafenib resistance PLC Xenograft
| Category | Sorafenib resistance PLC Xenograft |
|---|---|
| control | 720.0 |
| Sora | 765.0 |
| Exo-scramble siRNA+sora | 729.0 |
| Exo-siGRP78+sora
 | 90.0 |
### Chart:
| Category | Sorafenib sensitive PLC Xenograft |
|---|---|
| control | 450.0 |
| Sora | 180.0 |
| Exo-scramble siRNA+sora | 198.0 |
| Exo-siGRP78+sora
 | 0.0 |*
*
*
Tumor weight(mg)
